# Hemichannels contribute to mitochondrial Ca^2+^ and morphology alterations evoked by ethanol in astrocytes

**DOI:** 10.3389/fcell.2024.1434381

**Published:** 2024-07-26

**Authors:** Tanhia F. Alvear, Arantza Farias-Pasten, Sergio A. Vergara, Juan Prieto-Villalobos, Antonia Silva-Contreras, Fernando A. Fuenzalida, Rodrigo A. Quintanilla, Juan A. Orellana

**Affiliations:** ^1^ Departamento de Neurología, Escuela de Medicina and Centro Interdisciplinario de Neurociencias, Facultad de Medicina, Pontificia Universidad Católica de Chile, Santiago, Chile; ^2^ Laboratory of Neurodegenerative Diseases, Facultad de Ciencias de La Salud, Instituto de Ciencias Biomédicas, Universidad Autónoma de Chile, Santiago, Chile

**Keywords:** mitochondria, astrocyte, alcoholism, hemichannels, connexin 43, pannexin-1

## Abstract

Alcohol, a toxic and psychoactive substance with addictive properties, severely impacts life quality, leading to significant health, societal, and economic consequences. Its rapid passage across the blood-brain barrier directly affects different brain cells, including astrocytes. Our recent findings revealed the involvement of pannexin-1 (Panx1) and connexin-43 (Cx43) hemichannels in ethanol-induced astrocyte dysfunction and death. However, whether ethanol influences mitochondrial function and morphology in astrocytes, and the potential role of hemichannels in this process remains poorly understood. Here, we found that ethanol reduced basal mitochondrial Ca^2+^ but exacerbated thapsigargin-induced mitochondrial Ca^2+^ dynamics in a concentration-dependent manner, as evidenced by Rhod-2 time-lapse recordings. Similarly, ethanol-treated astrocytes displayed increased mitochondrial superoxide production, as indicated by MitoSox labeling. These effects coincided with reduced mitochondrial membrane potential and increased mitochondrial fragmentation, as determined by MitoRed CMXRos and MitoGreen quantification, respectively. Crucially, inhibiting both Cx43 and Panx1 hemichannels effectively prevented all ethanol-induced mitochondrial abnormalities in astrocytes. We speculate that exacerbated hemichannel activity evoked by ethanol may impair intracellular Ca^2+^ homeostasis, stressing mitochondrial Ca^2+^ with potentially damaging consequences for mitochondrial fusion and fission dynamics and astroglial bioenergetics.

## 1 Introduction

Alcohol is a toxic and psychoactive substance with addictive properties that significantly impair life quality with serious health, societal, and economic ramifications (Carvalho et al., 2019). The consumption of alcohol dates back to the origin of the human species (Sharma et al., 2010; Dudley and Maro, 2021). Indeed, our hunter-gatherer predecessors already consumed alcoholic beverages derived from the fermentation of fruits, grains, and even honey (Dudley, 2002). In contemporary societies, drinking is a routine part of the social landscape for many in the population (Sudhinaraset et al., 2016). This is particularly true for adolescents, where alcohol often accompanies socialization linked to anxiolytic effects and rewarding sensations (Scott et al., 2017; MacArthur et al., 2020). More relevantly, the link between alcohol consumption at a young age and the heightened likelihood of developing alcohol use disorders in adulthood is extensively documented (DeWit et al., 2000; Tavolacci et al., 2019; Creswell et al., 2022).

Much of alcohol’s effects on behavior stem from its ability to rapidly cross the blood-brain barrier and directly affect various types of brain cells (Crone, 1965; Egervari et al., 2021). In the brain, ethanol, the major molecular component of alcoholic beverages, causes diverse acute and short-term effects, such as dysmetria, slower reaction times, increased talkativeness, slowed thinking, altered cognitive processing and memory “blackouts” (White et al., 2000; Vonghia et al., 2008; Jung and Namkoong, 2014). On the other hand, long-lasting repercussions of alcohol consumption include a deterioration in executive function and progressive decline in working/implicit memory and associative learning (Cairney et al., 2007; Pitel et al., 2009; Loeber et al., 2010). Although some of these effects are due to ethanol’s direct impact on molecular targets in neurons (Abrahao et al., 2017), several studies have suggested that glial cells may also play a role in this phenomenon (Blanco and Guerri, 2007; Gonzalez and Salido, 2009). This holds true, especially in the case of astrocytes, which are affected by ethanol in multiple ways (Adermark and Bowers, 2016; Coulter et al., 2023). Astrocytes form local and far-reaching interconnected networks that anatomically and functionally communicate neuronal synapses with brain blood vessels (Giaume et al., 2010). Astrocytic processes, along with pre- and postsynaptic neuronal structures, form the “tripartite synapse” in a complex physical and functional interaction. Within this framework, astrocytes detect neuronal activity and locally release bioactive molecules known as “gliotransmitters” (such as glutamate, ATP, and D-serine) in a Ca^2+^-dependent manner (Perea et al., 2009). These messengers play a role in regulating cerebral blood flow and exchanging energy-rich metabolites (Iadecola and Nedergaard, 2007), contributing to the immune response and maintaining brain interstitial fluid homeostasis (Weber and Barros, 2015).

Multiple lines of evidence indicate that astrocytes provide antioxidant protection for neurons (Chen et al., 2001; Vargas and Johnson, 2009; Turati et al., 2020). The latter are particularly susceptible to oxidative damage (Wang and Michaelis, 2010), which can lead to reactive oxygen species (ROS)-dependent alterations in macromolecules like DNA and proteins, ultimately causing cellular dysfunction and progressive cell death (Chatgilialoglu and O’Neill, 2001; Moldogazieva et al., 2018). Astrocytes, however, possess elevated levels of antioxidant molecules and ROS-detoxifying enzymes, such as glutathione, superoxide dismutase, and glutathione peroxidase, enabling them to offer neuroprotection against harmful free radicals (Wilson, 1997). These protective properties of astrocytes primarily stem from the metabolism and dynamic processes of their mitochondria (Hertz et al., 2007; Stephen et al., 2014). Mitochondrial fusion is essential for mitigating stress by mixing components of partially damaged mitochondria, while fission facilitates the generation of new mitochondria and serves as quality control by removing injured mitochondria, thereby promoting apoptosis during significant cellular damage (Westermann, 2010). In astrocytes, the dynamics of mitochondrial fusion and fission influence energy metabolism and intracellular Ca^2+^ ([Ca^2+^]_i_) regulation (Stephen et al., 2014), both critical for maintaining proper astrocytic neuroprotective functions (Cabezas et al., 2012). Significantly, past research has documented that ethanol triggers mitochondrial ROS production in cultured astrocytes (Russo et al., 2001; Gonzalez et al., 2007; Alfonso-Loeches et al., 2014). Nevertheless, the mechanism driving this effect and whether other mitochondrial functions are affected in astrocytes exposed to ethanol are still unclear.

We recently found that ethanol triggers astroglial dysfunction and subsequent cell death through a series of inflammatory pathways, with a pivotal role played by the activation of hemichannels (Gomez et al., 2024). These channels consist of six connexin or seven pannexin monomers arranged around a central pore, enabling the exchange of ions and small molecules between the cytosol and extracellular space (Giaume et al., 2021). While both connexin and pannexin hemichannels are considered large-pore channels (Syrjanen et al., 2021), they vary significantly in terms of their amino acid sequence, pharmacological properties, conductance, selectivity, and posttranslational regulatory mechanisms (Ebihara et al., 2003; D’Hondt et al., 2013; Patel et al., 2014). Astrocytes predominantly express connexin 43 (Cx43) and pannexin-1 (Panx1) hemichannels, facilitating the release of gliotransmitters crucial for synaptic transmission, plasticity, behavior, and memory (Chever et al., 2014; Meunier et al., 2017; Cheung et al., 2022; Linsambarth et al., 2022; Vasile et al., 2022). In pathological conditions, the exacerbated activation of astrocytic hemichannels results in [Ca^2+^]_i_ overload, ionic imbalance, and the release of potentially excitotoxic molecules (Santiago et al., 2011; Wei et al., 2014; Garre et al., 2016; Chavez et al., 2019; Diaz et al., 2019). This contributes to the development and worsening of various brain diseases (Yi et al., 2016; Zhang et al., 2021; Almad et al., 2022; Guo et al., 2022). While past studies have shown that hemichannels influence mitochondrial function in both normal and diseased states (Gadicherla et al., 2017; Zhang et al., 2022; Su et al., 2023), it remains unclear whether hemichannels play a role in the potential mitochondrial changes induced by ethanol in astrocytes. Here, we found that ethanol enhances mitochondrial Ca^2+^ dynamics and boosts superoxide production while also disrupting mitochondrial membrane potential. These effects were associated with reduced mitochondrial footprint and network complexity accompanied of increased mitochondrial fragmentation. Critically, inhibiting both Cx43 and Panx1 hemichannels significantly prevented these ethanol-induced mitochondrial changes in astrocytes.

## 2 Material and methods

### 2.1 Reagents and antibodies

HEPES, DNAse I, poly-L-lysine and probenecid (Prob) were purchased from Sigma-Aldrich (St. Louis, MO, United States). Fetal bovine serum (FBS) was obtained from Hyclone (Logan, UT, United States). Penicillin, streptomycin, Trypsin 10X, Hank’s solution, ATP determination kit, Dulbecco’s Modified Eagle Medium (DMEM), Phosphate-Buffered Saline (PBS), MitoTracker™ Red CMXRos (CMXRos), Rhod-2 AM, MitoTracker™ Green FM (MitoGreen), MitoSOX™, ethidium (Etd) bromide (10 mg/mL) and Hoechst 33342 were purchased from Thermo Fisher Scientific (Waltham, MA, United States). Gap19 (KQIEIKKFK, intracellular loop domain of Cx43), and ^10^panx1 (WRQAAFVDSY, first extracellular loop domain of Panx1) peptides were obtained from Genscript (New Jersey, United States).

### 2.2 Animals

Animal experimentation was conducted in accordance with the guidelines for the care and use of experimental animals of the US National Institutes of Health (NIH), the *ad hoc* committee of the Chilean government (ANID), the Bioethics Committee of the Pontificia Universidad Católica de Chile (PUC) (No. 200605010) and the European Community Council Directives of 24th November 1986. C57BL/6 (PUC) mice of 8–9 weeks of age were housed in cages in a temperature-controlled (24°C) and humidity-controlled vivarium under a 12 h light/dark cycle (lights on 8:00 a.m.), with *ad libitum* access to food and water.

### 2.3 Cell cultures

Astroglial cell primary cultures were prepared from the cortex of postnatal day 2 (P2) mice as previously described (Avendano et al., 2015). Briefly, brains were removed, and cortices were dissected. Meninges were carefully peeled off and tissue was mechanically dissociated in Ca^2+^ and Mg^2+^ free Hank’s balanced salt solution (CM-HBSS) with 0.25% trypsin and 1% DNase. Cells were seeded onto glass coverslips (Fisher Scientific, Waltham, MA, United States) placed inside 12-well plastic plates (Corning, NY, United States) at the density of 3 × 10^5^ cells/well in DMEM, supplemented with penicillin (5 U/mL), streptomycin (5 μg/mL), and 10% FBS. Cells were grown at 37°C in a 5% CO_2_/95% air atmosphere at nearly 100% relative humidity. Following 8–10 days *in vitro* (DIV), 1 µM AraC was added for 3 days to suppress the proliferation of microglia. Medium was changed twice a week and cultures were used after 3 weeks.

### 2.4 Treatments

Astrocytes were treated for 0, 1, 24, 48 or 72 h with 100 mM of EtOH. The following pharmacological agents were pre-incubated 30 min prior and co-incubated with 100 mM ethanol before imaging acquisition: 100 µM gap19 (mimetic peptide against Cx43 hemichannels), 100 µM ^10^panx1 (mimetic peptide against Panx1 hemichannels) and 500 µM Probenecid (Panx1 hemichannel blocker). In addition, during time-lapse recordings of Rhod-2 or Etd fluorescence (see below), cells were acutely exposed to 3 µM thapsigargin.

### 2.5 Mitochondrial Ca^2+^ imaging

Astrocytes plated on glass coverslips were loaded with 1 µM Rhod-2 AM and 25 nM MitoGreen in DMEM without serum at 37°C for 30 min and then washed three times in Locke’s solution (154 mM NaCl, 5.4 mM KCl, 2.3 mM CaCl_2_, 5 mM HEPES, pH 7.4) followed by de-esterification at 37°C for 15 min. Rhod-2 and MitoGreen fluorescences were acquired using a Zeiss Axio Observer D.1 Inverted Microscope with an EC Plan-Neofluar 63x/1.25 Oil M27 immersion objective (Carl Zeiss AG, Zeiss, Oberkochen, Germany). Rhod-2 and MitoGreen were excited at 590 and 470 nm, respectively, with a Solid-State Colibri 7 LED illuminator (Carl Zeiss AG, Zeiss, Oberkochen, Germany), whereas emissions were recorded at 570 and 512 nm, respectively. Changes were monitored using an AxioCam MRm monochrome digital camera R3.0 (Carl Zeiss AG, Zeiss, Oberkochen, Germany), and Software ZEN Pro [Zen 2.3 (blue edition), Carl Zeiss AG, Oberkochen, Germany] for image acquisition. Time-lapse series were subsequently recorded every 60 s for 25 min. The Fiji-ImageJ software was used for offline image analysis (Schindelin et al., 2012). Raw MitoGreen images were converted into a mask by image binarization and then used to filter the raw fluorescence images for Rhod-2. This ensured that only overlapping regions were considered for further analysis. Regions of interest (ROIs) were then defined in the raw or filtered images and the average fluorescence intensity was calculated for each ROI at each time point with the following formula: Corrected total cell fluorescence (CTCF) = Integrated Density of selected cell – [(Area of selected cell) × (Mean Gray Value of background)]. The parameters above were measured using the “Measure” tab in ImageJ software and are detailed as follows: Integrated Density: The product of Area and Mean Gray Value; Area: The area of the selection in square pixels; Mean Gray Value: The sum of the gray values of all the pixels in the selection divided by the number of pixels. The CTCF formula determines the total cell fluorescence intensity by subtracting the background fluorescence and correcting for intensity differences due to area. Subsequently, the ROI data were normalized relative to its starting fluorescence using Microsoft Excel (Seattle, WA, United States), employing the formula F/F0, where F represented the intensity of fluorescence emission recorded during the experiment and F0 represented the fluorescence intensity at the start of the experiment. The averaged fluorescence values were plotted using GraphPad Prism 7 software (La Jolla, California, United States). The area under the curve (AUC) of Rhod-2 fluorescence over time, along with the maximal Rhod-2 fluorescence value, were also determined.

### 2.6 Mitochondrial superoxide production

Astrocytes plated on glass coverslips were loaded with 0.8 µM Mitosox in DMEM without serum at 37°C for 15 min and then washed three times in Locke’s solution. Afterwards, cells were loaded with 25 nM MitoGreen in DMEM without serum at 37°C for 15 min and washed three times in Locke’s solution followed by de-esterification at 37°C for 15 min. MitoSOX and MitoGreen fluorescences were acquired using a Zeiss Axio Observer D.1 Inverted Microscope with an EC Plan-Neofluar 63x/1.25 Oil M27 immersion objective (Carl Zeiss AG, Zeiss, Oberkochen, Germany). MitoSOX and MitoGreen were excited at 590 and 470 nm, respectively, with a Solid-State Colibri 7 LED illuminator (Carl Zeiss AG, Zeiss, Oberkochen, Germany), whereas emissions were recorded at 570 and 512 nm, respectively. Changes were monitored using an AxioCam MRm monochrome digital camera R3.0 (Carl Zeiss AG, Zeiss, Oberkochen, Germany), and Software ZEN Pro (Zen 2.3 [blue edition], Carl Zeiss AG, Oberkochen, Germany) for image acquisition. A minimum of 10 cells were analyzed for each coverslip. The Fiji-ImageJ software was used for offline image analysis (Schindelin et al., 2012). Raw MitoGreen images were converted into a mask by image binarization and then used to filter the raw fluorescence images for MitoSOX. This ensured that only overlapping regions were considered for further analysis. Regions of interest (ROIs) were then defined in the raw or filtered images, and the average fluorescence intensity was calculated for each ROI using the CTCF formula described above. The averaged fluorescence values were plotted using GraphPad Prism 7 software (La Jolla, California, United States).

### 2.7 Mitochondrial network analysis

The Fiji-ImageJ software (Schindelin et al., 2012) was used for offline image analysis of MitoGreen fluorescence obtained from the abovementioned experiments. Cells were selected and cut with the crop tool to facilitate their analysis when they fulfilled the following criteria: 1) consistent and strong staining along the entire mitochondrial area, and 2) relative isolation from neighboring cells to avoid overlap. The analysis of mitochondrial networks was performed using the macro tool for Fiji-ImageJ MiNA (Valente et al., 2017). Before using MiNA, the following preprocessing steps of Fiji-ImageJ tools were done: “Unsharp Mask,” “Enhance Local Contrast (CLAHE),” and “Median Filters” to enhance the edges, sharpen the images with unchanged contrast, equalize histograms locally with limited ranges of changes to avoid overamplifying noise, and eliminate salt-and-pepper noise. Then, in the MiNA pipeline, images were converted into binary (black-and-white) images through the default thresholding method (IsoData algorithm of ImageJ) and then transformed into skeletons, a framework representing lines with a one-pixel width, using the default thinning algorithm. Afterward, the following network parameters were measured: mitochondrial footprint (area of the image consumed by mitochondrial fluorescence), branch length mean (length of all the lines used to represent the mitochondrial structures), summed branch length mean (the sum of all branch lengths divided by the number of independent skeletons) and number of networks (the number of attached lines used to represent each structure). The mean values were plotted using GraphPad Prism 7 software (La Jolla, California, United States).

### 2.8 Mitochondrial fragmentation analysis

The Fiji-ImageJ software (Schindelin et al., 2012) was used for offline image analysis of MitoGreen fluorescence obtained from the abovementioned experiments. Cells were selected for analysis using the same criteria mentioned in the previous section. Before image analysis, the following preprocessing steps of Fiji-ImageJ tools were done: “Unsharp Mask,” “Enhance Local Contrast (CLAHE),” and “Median Filters” to enhance the edges, sharpen the images with unchanged contrast, equalize histograms locally with limited ranges of changes to avoid overamplifying noise, and eliminate salt-and-pepper noise. Then, images were converted into binary (black-and-white) images through the default thresholding method (IsoData algorithm of ImageJ) and the particle analysis command was used. The following parameters related to the mitochondria count and profile were measured: “Count,” “Area,” “Perimeter,” “Form factor,” and “Aspect ratio.” In addition, we calculated the mitochondrial fragmentation count (MFC) using the number of black pixels comprising binary mitochondrial networks using the Histogram function within ImageJ. The MFC is calculated by dividing the number of discrete mitochondrial segments in the cell by the total mitochondrial mass (white pixels) and arbitrarily multiplying by 1,000 (Rehman et al., 2012). The mean values were plotted using GraphPad Prism 7 software (La Jolla, California, United States).

### 2.9 Mitochondrial membrane potential

Astrocytes plated on glass coverslips were loaded with 25 nM µM CMXRos and 25 nM MitoGreen in DMEM without serum at 37°C for 30 min and then washed three times in Locke’s solution followed by de-esterification at 37°C for 15 min. CMXRos and MitoGreen fluorescences were acquired using a Zeiss Axio Observer D.1 Inverted Microscope with an EC Plan-Neofluar 63x/1.25 Oil M27 immersion objective (Carl Zeiss AG, Zeiss, Oberkochen, Germany). CMXRos and MitoGreen were excited at 590 and 470 nm, respectively, with a Solid-State Colibri 7 LED illuminator (Carl Zeiss AG, Zeiss, Oberkochen, Germany), whereas emissions were recorded at 570 and 512 nm, respectively. Changes were monitored using an AxioCam MRm monochrome digital camera R3.0 (Carl Zeiss AG, Zeiss, Oberkochen, Germany), and Software ZEN Pro [Zen 2.3 (blue edition), Carl Zeiss AG, Oberkochen, Germany] for image acquisition. A minimum of 10 cells were analyzed for each coverslip. The Fiji-ImageJ software was used for offline image analysis (Schindelin et al., 2012). Raw MitoGreen images were converted into a mask by image binarization and then used to filter the raw fluorescence images for CMXRos. This ensured that only overlapping regions were considered for further analysis. Regions of interest (ROIs) were then defined in the raw or filtered images, and the average fluorescence intensity was calculated for each ROI using the CTCF formula described above. The averaged fluorescence values of CMXRos/MitoGreen ratio were plotted using GraphPad Prism 7 software (La Jolla, California, United States).

### 2.10 Dye uptake

For dye uptake experiments in astrocytes, they were plated on 12 mm glass coverslips and, after 2 weeks of culture, were washed twice in Hank’s balanced salt solution. Then, astrocytes were incubated at room temperature with recording solution (in mM): 148 NaCl, 5 KCl, 1.8 CaCl_2_, 1 MgCl_2_, 5 glucose, and 5 HEPES, pH 7.4, containing 5 μM Etd and mounted on the stage of a Zeiss Axio Observer D.1 Inverted Microscope with an EC Plan-Neofluar 63x/1.25 Oil M27 immersion objective (Carl Zeiss AG, Zeiss, Oberkochen, Germany). Rhod-2 and MitoGreen were excited at 590 and 470 nm, respectively, with a Solid-State Colibri 7 LED illuminator (Carl Zeiss AG, Zeiss, Oberkochen, Germany), whereas emissions were recorded at 570 and 512 nm, respectively. Changes were monitored using an AxioCam MRm monochrome digital camera R3.0 (Carl Zeiss AG, Zeiss, Oberkochen, Germany), and Software ZEN Pro [Zen 2.3 (blue edition), Carl Zeiss AG, Oberkochen, Germany] for image acquisition. Time-lapse series were subsequently recorded every 60 s for 25 min. The Fiji-ImageJ software was used for offline image analysis (Schindelin et al., 2012). The average fluorescence intensity was calculated for each ROI at each time point with the CTCF formula described above. The mean slope of the relationship over a given time interval (ΔF/ΔT) represents the dye uptake rate and was calculated with regression lines that were fitted to points before and after the various experimental conditions using Microsoft Excel (Seattle, WA, United States). The mean values of slopes were plotted using GraphPad Prism 7 software (La Jolla, California, United States) and expressed as AU/min.

### 2.11 Data analysis and statistics

Detailed statistical results were included in the figure legends. Statistical analyses were performed using GraphPad Prism (version 7, GraphPad Software, La Jolla, CA). Normality and equal variances were assessed using the Shapiro-Wilk normality test and Brown-Forsythe test, respectively. Unless otherwise stated, data that passed these tests were analyzed by unpaired t-test in case of comparing two groups, whereas in case of multiple comparisons, data were analyzed by one or two-way analysis of variance (ANOVA) followed, in case of significance, by a Tukey’s *post hoc* test. A probability of *p* < 0.05 was considered statistically significant.

## 3 Results

### 3.1 Ethanol boosts thapsigargin-induced mitochondrial Ca^2+^ dynamics via Panx1 and Cx43 hemichannels in astrocytes

Mitochondrial Ca^2+^ plays a crucial role in regulating the function of astrocytes under both physiological and pathophysiological conditions. Ethanol has been shown to influence mitochondrial function in astrocytes, as evidenced by its stimulating effect on mitochondrial ROS production (Gonzalez et al., 2007; Alfonso-Loeches et al., 2014). Based on these findings and the understanding that hemichannels regulate mitochondrial function in both normal and diseased states (Gadicherla et al., 2017; Zhang et al., 2022; Su et al., 2023), we explored whether ethanol affects mitochondrial Ca^2+^ levels (Ca^2+^_m_), in astrocytes and, if so, whether hemichannels play a role in this process. To monitor Ca^2+^_m_, we loaded astrocytes with the fluorescent membrane-permeable dye Rhod-2. This dye, possessing a positive charge, accumulates in the mitochondrial matrix driven by the mitochondrial membrane potential (ΔΨ_m_) according to the Nernst equation (Trollinger et al., 1997). Raw fluorescence images for Rhod-2 were filtered through an image binarization mask of the fluorescence of MitoTracker™ Green FM (MitoGreen), a prototypical mitochondrial probe. This ensured that Rhod-2 fluorescence was specifically measured in mitochondrial regions.

**FIGURE 1:**
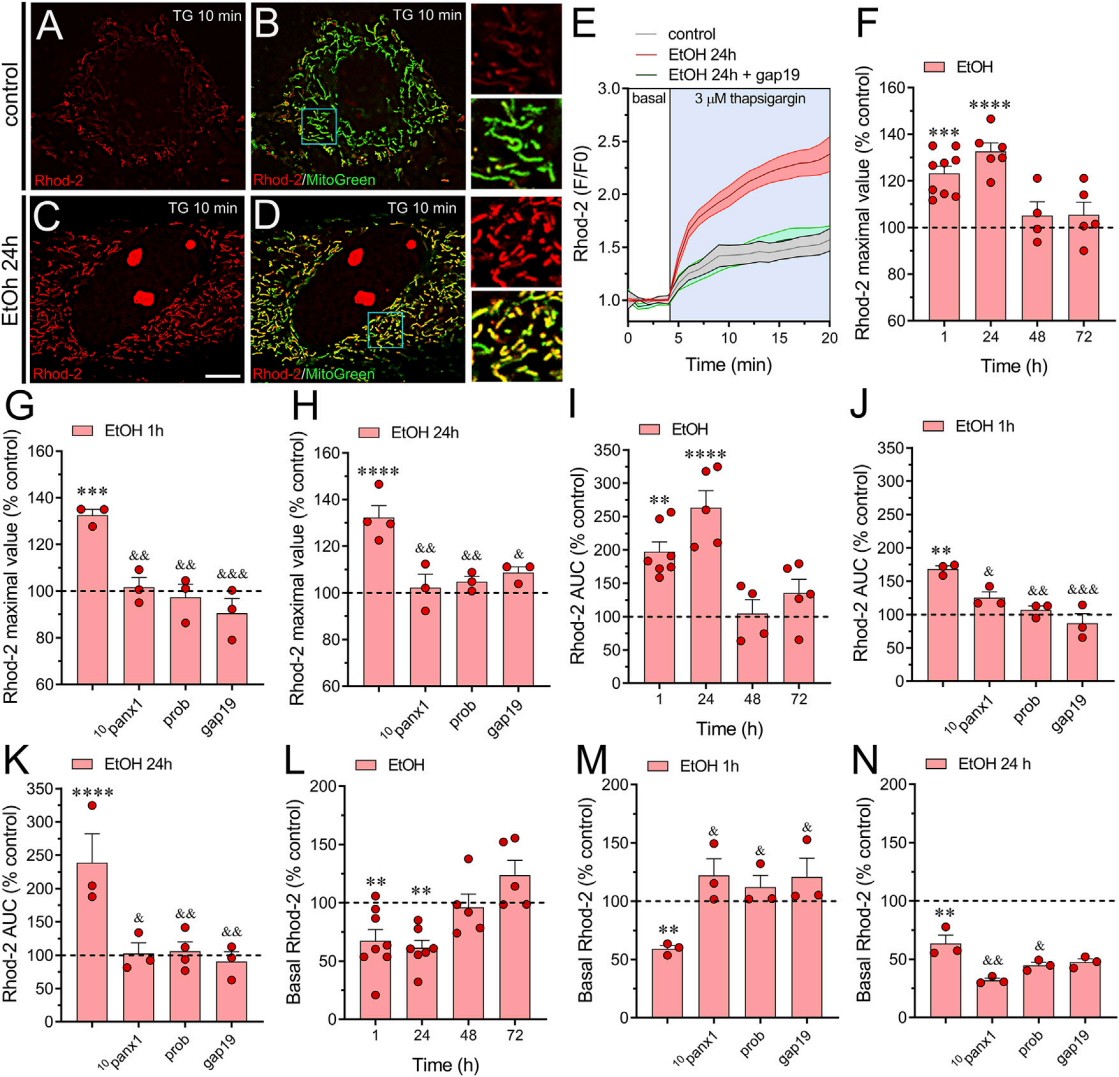
Panx1 and Cx43 hemichannels contribute to the increased thapsigargin-induced mitochondrial Ca^2+^ dynamics evoked by ethanol in astrocytes. **(A–D)** Representative photomicrographs of Rhod-2 (red) and MitoGreen (green) fluorescence after 10 min of stimulation with 3 µM thapsigargin (TG) by astrocytes under control conditions **(A, B)** or following the treatment with 100 mM ethanol for 24 h **(C, D)**. Insets: × 2.5 magnification of the indicated area of panels **(B, D)**. **(E)** Representative plots of Rhod-2 fluorescence in basal conditions or after acute stimulation with 3 µM thapsigargin (light blue box) by astrocytes under control conditions (gray line) or following 24 h of treatment with 100 mM ethanol (red line) alone or in combination with 100 µM gap19 (green line). **(F)** Averaged data normalized to control conditions (dashed line) of thapsigargin-induced maximal Rhod-2 fluorescence value by astrocytes treated with 100 mM ethanol for several periods. **(G, H)** Averaged data normalized to control conditions (dashed line) of thapsigargin-induced maximal Rhod-2 fluorescence value by astrocytes treated for 1 h **(G)** or 24 h **(H)** with 100 mM ethanol alone or plus the following pharmacological agents: 100 µM ^10^panx1, 500 µM probenecid (Prob) or 100 µM gap19. **(I)** Averaged data normalized to control conditions (dashed line) of thapsigargin-induced area under the curve (AUC) of Rhod-2 fluorescence over time by astrocytes treated with 100 mM ethanol for several periods. **(J, K)** Averaged data normalized to control conditions (dashed line) of thapsigargin-induced AUC of Rhod-2 fluorescence over time by astrocytes treated for 1 h **(J)** or 24 h **(K)** with 100 mM ethanol alone or plus the following pharmacological agents: 100 µM ^10^panx1, 500 µM probenecid (Prob) or 100 µM gap19. **(L)** Averaged data normalized to control conditions (dashed line) of basal Rhod-2 fluorescence by astrocytes treated with 100 mM ethanol for several periods. **(M, N)** Averaged data normalized to control conditions (dashed line) of basal Rhod-2 fluorescence by astrocytes treated for 1 h **(M)** or 24 h **(N)** with 100 mM ethanol alone or plus the following pharmacological agents: 100 µM ^10^panx1, 500 µM probenecid (Prob) or 100 µM gap19. **p < 0.01, ***p < 0.001, ****p < 0.0001; ethanol compared to control conditions; ^&^ *p* < 0.05, ^&&^ *p* < 0.01; ^&&&^ *p* < 0.001; effect of pharmacological agents compared to ethanol treatment (one-way ANOVA followed by Tukey’s *post hoc* test). Each dot represents an independent culture experiment from a different litter of animals. The value reflects the mean of at least three coverslip replicates, with a minimum of five cells analyzed per coverslip. Calibration bar: 7 μm.

**FIGURE 2:**
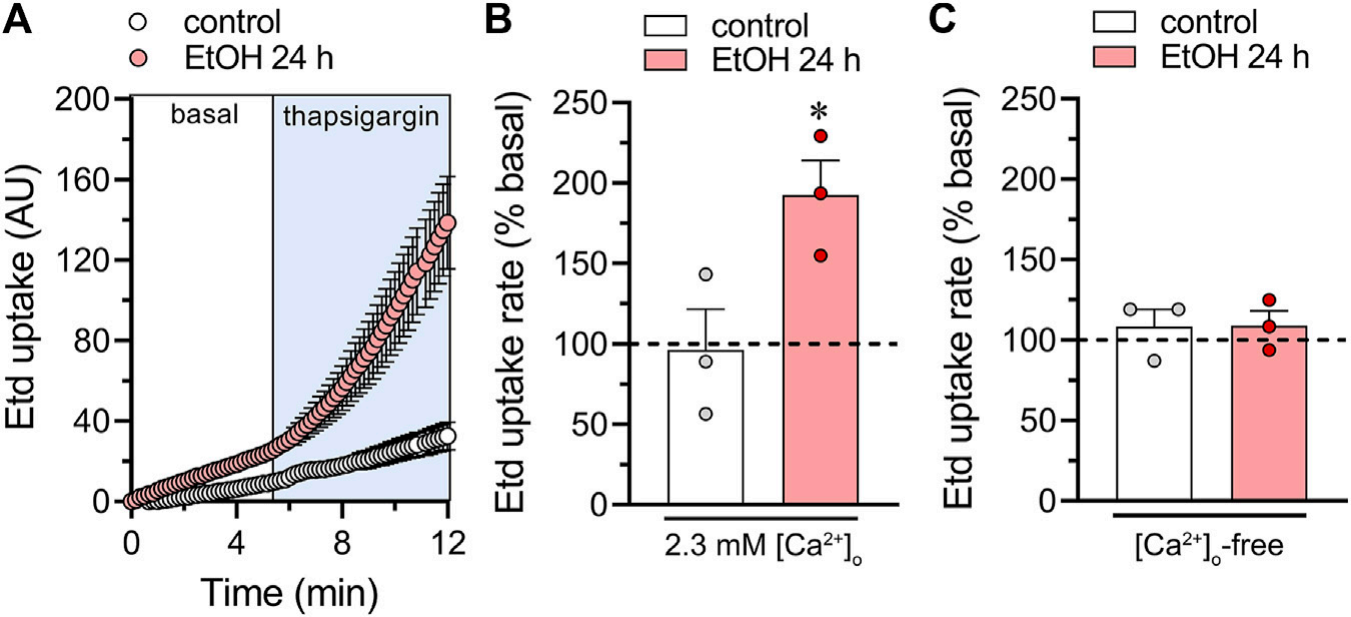
Acute thapsigargin exposure increases the activity of hemichannels in cultured astrocytes. **(A)** Time-lapse measurements of Etd uptake under basal conditions (white box) or upon the acute treatment with 3 µM thapsigargin (ligh blue box) by astrocytes under control conditions (white circles) or following 24 h of treatment with 100 mM ethanol (red circles). **(B, C)** Averaged Etd uptake rate normalized with the basal condition (dashed line) of the acute effect of 3 µM thapsigargin on astrocytes under control conditions (white bars) or following 24 h of treatment with 100 mM ethanol (red bars). The effect of thapsigargin is shown with bath recording solution containing either normal extracellular Ca^2+^ **(B)** or zero Ca^2+^ **(C)**. *p < 0.05, ethanol treatment compared to control conditions (two-tailed Student’s unpaired test). Each dot represents an independent culture experiment from a different litter of animals. The value reflects the mean of at least three coverslip replicates, with a minimum of ten cells analyzed per coverslip.

To assess whether ethanol plays a role in maintaining Ca^2+^_m_ homeostasis in astrocytes, we elevated [Ca^2+^]_i_ by exposing cells to thapsigargin. Thapsigargin induces an increase in [Ca^2+^]_i_ by depleting Ca^2+^ stores in the endoplasmic reticulum (ER) and inhibiting the flux of Ca^2+^ to the ER through the inhibition of sarcoplasmic/endoplasmic reticular Ca^2+^ ATPase (SERCA) (Rogers et al., 1995). By using thapsigargin treatment, we established experimental conditions that allow us to specifically examine the forced Ca^2+^ entry into the mitochondria as a readout of Ca^2+^_m_ homeostasis. Time-lapse recordings of Rhod-2 showed that when control astrocytes were acutely stimulated with 3 µM thapsigargin, there was a large and persistent increase in Ca^2+^_m_ (Figures 1A, B, E). The level of Ca^2+^ within mitochondria remained elevated for more than 15 min after thapsigargin application (Figure 1E). To investigate the potential influence of ethanol on this phenomenon, we exposed astrocytes to ethanol. Previously, we demonstrated that ethanol activates astroglial hemichannels rapidly within 1 h, with this activation persisting for 24–72 h at concentrations ranging from 10 to 100 mM (Gomez et al., 2024). Given that the maximal effect was observed at the highest concentration, 100 mM, we selected it to conduct the experiments in this work. Notably, astrocytes stimulated with ethanol exhibited a time-dependent increase in the maximum value of Ca^2+^_m_ response, peaking at higher levels after 1–24 h of treatment (Figures 1C–F). This phenomenon was accompanied by a time-dependent elevation in the area under the curve (AUC) of thapsigargin-induced Ca^2+^_m_ response (Figure 1I). The contribution of hemichannels to the thapsigargin-mediated increase in Ca^2+^_m_ evoked by ethanol was explored using ^10^panx1 and gap19, two mimetic peptides that inhibit Panx1 or Cx43 hemichannels by interacting with the first extracellular loop of Panx1 (Pelegrin and Surprenant, 2006) or the intracellular L2 loop of Cx43 (Iyyathurai et al., 2013), respectively. ^10^panx1 (100 µM) totally prevented the thapsigargin-mediated increase in Ca^2+^_m_ evoked by treatment with ethanol for 1 or 24 h (Figures 1G, H, J, K). Similar preventive effects on ethanol-induced boost on Ca^2+^_m_ dynamics were observed upon blockade of Panx1 hemichannels with probenecid (Figures 1G, H, J, K), a well-known pharmacological inhibitor of these channels (Silverman et al., 2008). In the same line, we observed that gap19 (100 µM) elicited a potent inhibitory response on thapsigargin-mediated increase in Ca^2+^_m_ evoked by ethanol (Figures 1G, H, J, K). Notably, ethanol also reduced basal Ca^2+^_m_ after 1 or 24 h of treatment (Figure 1M). Interestingly, blocking Cx43 or Panx1 hemichannels prevented the decrease in basal Ca^2+^_m_ evoked by 1 h but not 24 h of ethanol treatment (Figures 1L–N). In fact, blocking Panx1 hemichannels during the 24-hour ethanol treatment further accentuated the reduction in basal Ca^2+^_m_ compared to the ethanol treatment alone (Figure 1N). Cx43 and Panx1 hemichannels are permeable to Ca^2+^, and an increase in [Ca^2+^]_i_ leads to their activation (De Bock et al., 2012; Fiori et al., 2012; Lopez et al., 2021; Salgado et al., 2024). Given that is thapsigargin is a well-known agent that increases in [Ca^2+^]_i_, we next determined whether hemichannel opening occurs during thapsigargin application. For that, we performed time-lapse recordings of ethidium (Etd) uptake under basal conditions and upon acute thapsigargin stimulation. Etd is a dye that enters the cytoplasm of healthy cells through hemichannels (Johnson et al., 2016). Upon intercalating with base pairs of DNA and RNA, Etd becomes fluorescent, indicating hemichannel activity. We found that thapsigargin induced a significant increase in Etd uptake compared to basal conditions in astrocytes treated for 24 h with ethanol but not in control astrocytes (Figures 2A, B). Notably, when experiments were conducted in the absence of extracellular Ca^2+^, the thapsigargin-induced increase in Etd uptake did not occur (Figures 2A–C), indicating that Ca^2+^ entry from the extracellular space is crucial for this phenomenon. Together, these findings suggest that the activation of Cx43 and Panx1 hemichannels contributes to ethanol-induced alterations in basal Ca^2+^_m_ and thapsigargin-mediated Ca^2+^_m_ dynamics in astrocytes.

### 3.2 Panx1 and Cx43 hemichannels contribute to ethanol-induced increase in mitochondrial superoxide in astrocytes

**FIGURE 3:**
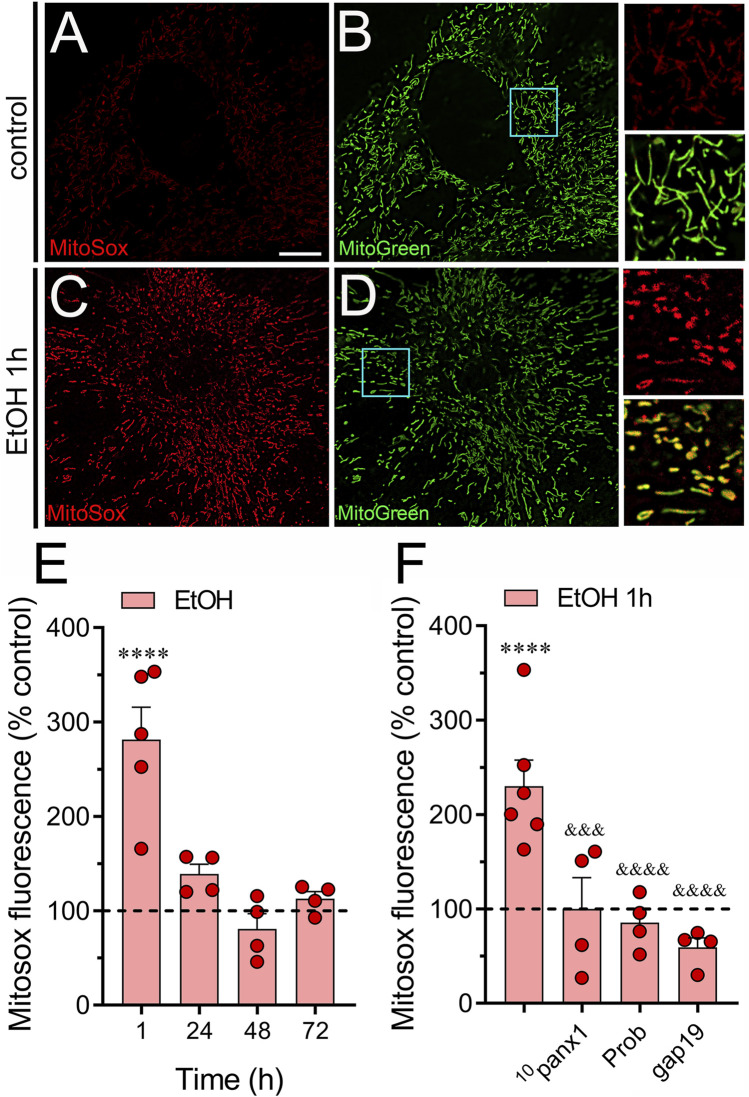
Ethanol augments the production of mitochondrial superoxide via the activation of Panx1 and Cx43 hemichannels in astrocytes. **(A–D)** Representative photomicrographs of MitoSox (red) and MitoGreen (green) fluorescence by astrocytes under control conditions **(A, B)** or following the treatment with 100 mM ethanol for 1 h **(C, D)**. Insets: × 2.5 magnification of the indicated area of panels **(B, D)**. **(E)** Averaged data normalized to control conditions (dashed line) of MitoSox fluorescence by astrocytes treated with 100 mM ethanol for several time periods. **(F)** Averaged data normalized to control conditions (dashed line) of MitoSox by astrocytes treated for 1 h with 100 mM ethanol alone or plus the following pharmacological agents: 100 µM ^10^panx1, 500 µM probenecid (Prob) or 100 µM gap19. ****p < 0.0001; ethanol compared to control conditions; ^&&&^ *p* < 0.001; ^&&&&^ *p* < 0.0001; effect of pharmacological agents compared to ethanol treatment (one-way ANOVA followed by Tukey’s *post hoc* test). Each dot represents an independent culture experiment from a different litter of animals. The value reflects the mean of at least three coverslip replicates, with a minimum of 60 cells analyzed per coverslip. Calibration bar: 10 μm.

Given that ethanol can elevate mitochondrial ROS production in cultured astrocytes (Gonzalez et al., 2007; Alfonso-Loeches et al., 2014), we investigated whether ethanol could influence mitochondrial superoxide production via the activation of hemichannels, known to alter intracellular oxidative status (Chi et al., 2016; Xu et al., 2018; Ma et al., 2020). The raw fluorescence images of MitoSOX were filtered using an image binarization mask derived from MitoGreen, guaranteeing that the Rhod-2 fluorescence was specifically measured in mitochondrial regions. Control astrocytes exhibited minimal superoxide fluorescence (Figures 3A, B), whereas those stimulated with ethanol for 1 h showed a roughly 4-fold increase in superoxide production (Figures 3C–E). This effect was transient, as longer ethanol treatments did not alter superoxide production compared to control levels (Figures 3C–E). Importantly, both ^10^panx1 and probenecid fully attenuated the increase in superoxide levels induced by 1-hour ethanol treatment (Figure 3F). Similarly, inhibition of Cx43 hemichannels with gap19 completely suppressed ethanol-induced mitochondrial superoxide production (Figure 3F). Overall, these findings underscore the involvement of Panx1 and Cx43 hemichannels in the heightened production of mitochondrial superoxide triggered by ethanol in astrocytes.

### 3.3 Ethanol disturbs mitochondrial morphology in astrocytes by a mechanism involving the activation of Panx1 and Cx43 hemichannels

**FIGURE 4:**
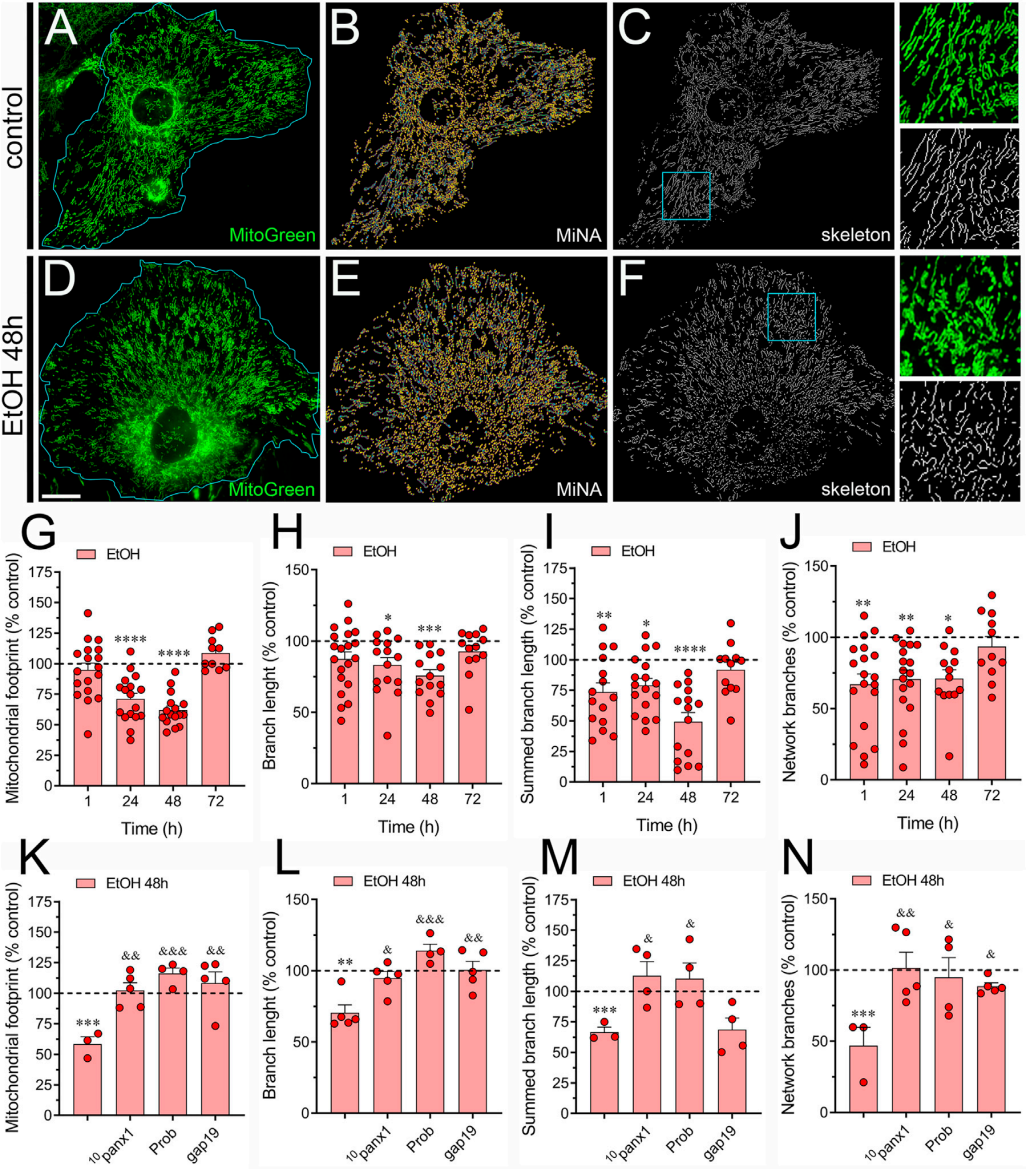
Panx1 and Cx43 hemichannels contribute to the ethanol-induced alterations in mitochondrial network morphology in astrocytes. **(A–F)** Representative photomicrographs of MitoGreen (green) fluorescence, segmented MitoGreen fluorescence with MiNa software and skeletonized MitoGreen fluorescence by astrocytes under control conditions **(A–C)** or following the treatment with 100 mM ethanol for 48 h **(D–F)**. Insets: × 2.5 magnification of the indicated area of panels **(C, F)**. **(G–J)** Averaged data normalized to control conditions (dashed line) of mitochondrial footprint **(G)**, branch length **(H)**, summed branch length **(I)** and number of network branches **(J)** by astrocytes treated with 100 mM ethanol for several time periods. **(K–N)** Averaged data normalized to control conditions (dashed line) of mitochondrial footprint **(G)**, branch length **(H)**, summed branch length **(I)** and number of network branches **(J)** by astrocytes treated for 48 h with 100 mM ethanol alone or plus the following pharmacological agents: 100 µM ^10^panx1, 500 µM probenecid (Prob) or 100 µM gap19. *p < 0.05, **p < 0.01, ***p < 0.001, ****p < 0.0001; ethanol compared to control conditions; ^&^ *p* < 0.05, ^&&^ *p* < 0.01; ^&&&^ *p* < 0.001; effect of pharmacological agents compared to ethanol treatment (one-way ANOVA followed by Tukey’s *post hoc* test). Each dot represents an independent culture experiment from a different litter of animals. The value reflects the mean of at least three coverslip replicates, with a minimum of 60 cells analyzed per coverslip. Calibration bar: 30 μm.

Mitochondria are vital cellular organelles known for their dynamic nature and multifunctionality, playing a key role in ATP production and various cellular processes (Quintana-Cabrera and Scorrano, 2023). Their dynamic behavior allows them to adapt their shape and structure to the cell’s physiological requirements, involving a balance between two processes: fission and fusion (Adebayo et al., 2021). These processes regulate mitochondrial size, shape, number, and distribution under normal and disease conditions (Chan, 2020). Although ethanol’s disruption of mitochondrial morphology in various cell types is well-documented (Bonet-Ponce et al., 2015; De Filippis et al., 2016), its specific impact on astrocytic mitochondria has yet to be investigated. Considering this, we focused on determining whether ethanol impacts mitochondrial network morphology in astrocytes using the MiNa plugin in the Fiji-ImageJ software (Valente et al., 2017). Upon visual inspection of the mitochondrial network labeled with MitoGreen, we observed a time-dependent reduction in the mitochondrial area or footprint by astrocytes treated with ethanol, peaking around 48 h of treatment (Figures 4A–G). A similar time-dependent decrease was found in the length of mitochondrial branches and summed mitochondrial branch length by ethanol-stimulated astrocytes (Figures 4H, I). Likewise, treatment with ethanol for 1 h diminished the number of mitochondrial network branches in astrocytes, returning to control levels after 72 h of treatment (Figure 4J). Notably, the pharmacological inhibition of Panx1 hemichannels or Cx43 hemichannels completely abolished the ethanol-induced reductions in mitochondrial footprint, mitochondrial branch length, summed mitochondrial branch length and mitochondrial network branches (Figures 4K–N). In summary, these observations suggest that ethanol reduces mitochondrial complexity in astrocytes by activating Panx1 and Cx43 hemichannels.

**FIGURE 5:**
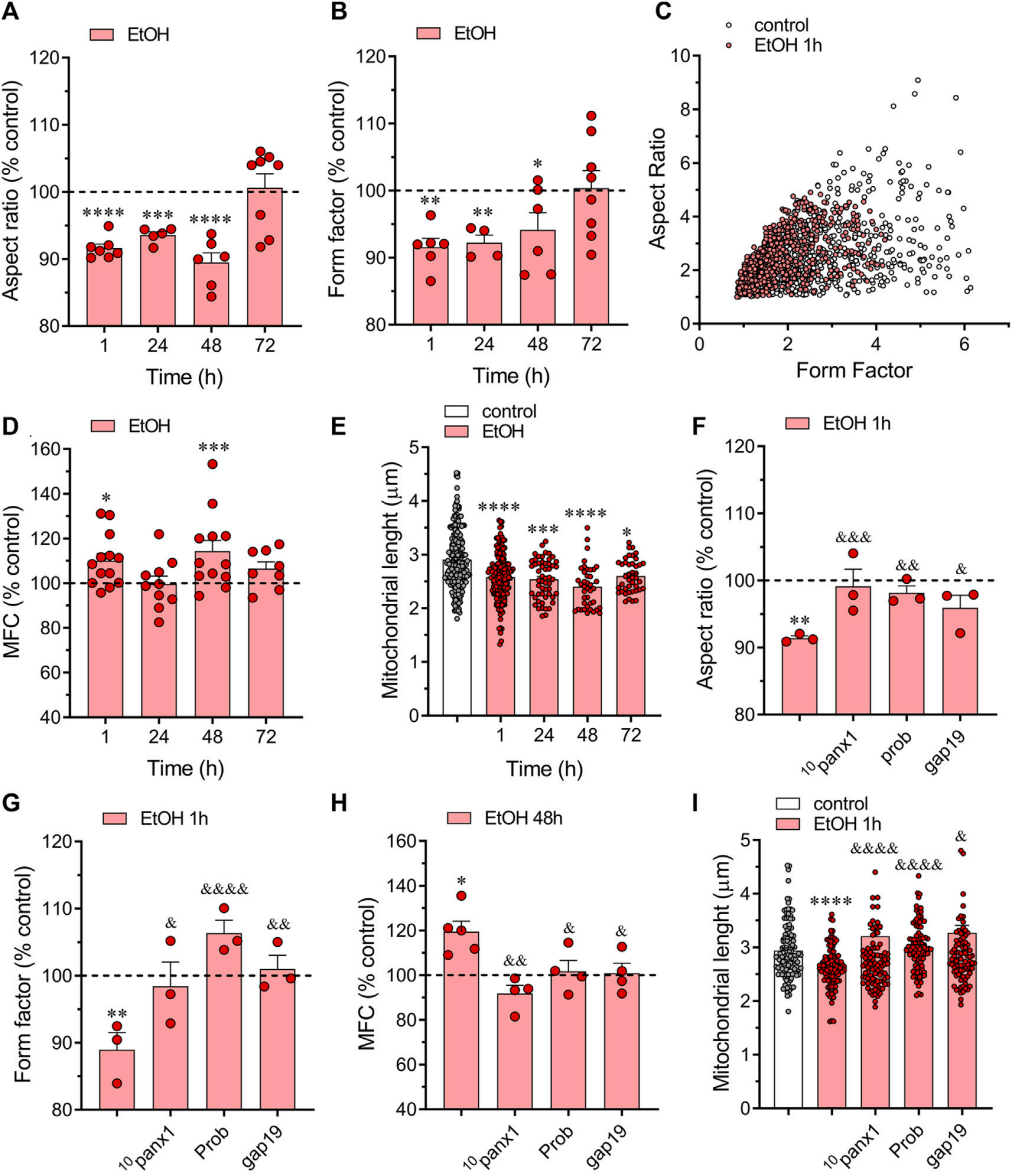
Panx1 and Cx43 hemichannels contribute to the ethanol-induced mitochondrial fragmentation in astrocytes. **(A, B)** Averaged data normalized to control conditions (dashed line) of aspect ratio **(A)** and form factor **(B)** by astrocytes treated with 100 mM ethanol for several time periods. **(C)** Scatter plot of form indexes distribution of individual mitochondria from a representative control (white circles) and 100 mM ethanol-treated astrocyte for 1 h (red circles). **(D)** Averaged data normalized to control conditions (dashed line) of mitochondrial fragmentation count (MFC) by astrocytes treated with 100 mM ethanol for several time periods. **(E)** Averaged data of mitochondrial length by astrocytes under control conditions (white violin) or treated with 100 mM ethanol for several time periods (red violins). Each dot represents the cell-averaged mitochondrial length of 20 mitochondria per cell. Overall, these dots encompass at least five independent culture experiments from different litters of animals. **(F–H)** Averaged data normalized to control conditions (dashed line) of aspect ratio **(F)**, form factor **(G)**, and MFC **(H)** by astrocytes treated for 48 h with 100 mM ethanol alone or plus the following pharmacological agents: 100 µM ^10^panx1, 500 µM probenecid (Prob) or 100 µM gap19. **(I)** Averaged data of mitochondrial length by astrocytes under control conditions (white violin) or treated for 48 h with 100 mM ethanol alone (red violin) or plus the following pharmacological agents: 100 µM ^10^panx1, 500 µM probenecid (Prob) or 100 µM gap19. *p < 0.05, **p < 0.01, ***p < 0.001, ****p < 0.0001; ethanol compared to control conditions; ^&^ *p* < 0.05, ^&&^ *p* < 0.01; ^&&&^ *p* < 0.001, ^&&&&^ *p* < 0.0001; effect of pharmacological agents compared to ethanol treatment (one-way ANOVA followed by Tukey’s *post hoc* test). Each dot represents an independent culture experiment from a different litter of animals. The value reflects the mean of at least three coverslip replicates, with a minimum of 60 cells analyzed per coverslip.

To assess mitochondrial fragmentation and elongation, we quantified the aspect ratio (AR) of mitochondria labeled with MitoGreen. The AR is calculated by measuring the long axis over the short axis. Thus, a higher AR value suggests a more elongated mitochondrion, while a lower AR value indicates mitochondrial fragmentation (Marchi et al., 2017). Treatment with ethanol for 1 h significantly decreased the AR in astrocytes, with this effect gradually returning to control values over time (Figure 5A). Next, we examined individual mitochondria using a form factor (FF). Mitochondrial FF indicates mitochondrial branching, with lower values suggesting a more spherical shape and higher values indicating highly interconnected or complex mitochondria (Marchi et al., 2017). As expected, astrocytes treated with ethanol exhibited a decreased FF compared to control conditions, peaking around 1 h of treatment and gradually returning to baseline over time (Figure 5B). These findings indicate that ethanol alters the morphological characteristics of astroglial mitochondria from elongated to a more fragmented phenotype. This notion was further supported by the distinct distribution of AR/FF between control and ethanol-treated astrocytes (Figure 5C).

We analyzed the mitochondrial fragmentation count (MFC) to further confirm that ethanol elicited mitochondrial fragmentation. This index is calculated by dividing the number of discrete mitochondrial segments by the total mitochondrial mass (Rehman et al., 2012). A higher MFC value corresponds to a more fragmented mitochondrial network. As expected, ethanol caused a significant increase in MCF compared to control conditions, this response being more prominent following 48 h of treatment with ethanol (Figure 5D). Moreover, astrocytes treated with ethanol exhibited a notable reduction in mitochondrial length following just 1 h of treatment, a change that persisted even after 72 h of exposure to ethanol (Figure 5E). Notably, using ^10^panx1, probenecid or gap19 almost completely prevented the ethanol-mediated alterations in morphological parameters and shape descriptors by astrocytes (Figures 5F–I). Altogether, these data indicate that ethanol induces mitochondrial fragmentation in astrocytes via the activation of Panx1 and Cx43 hemichannels.

### 3.4 Inhibition of hemichannels ameliorates the ethanol-induced decrease in mitochondrial membrane potential in astrocytes

**FIGURE 6:**
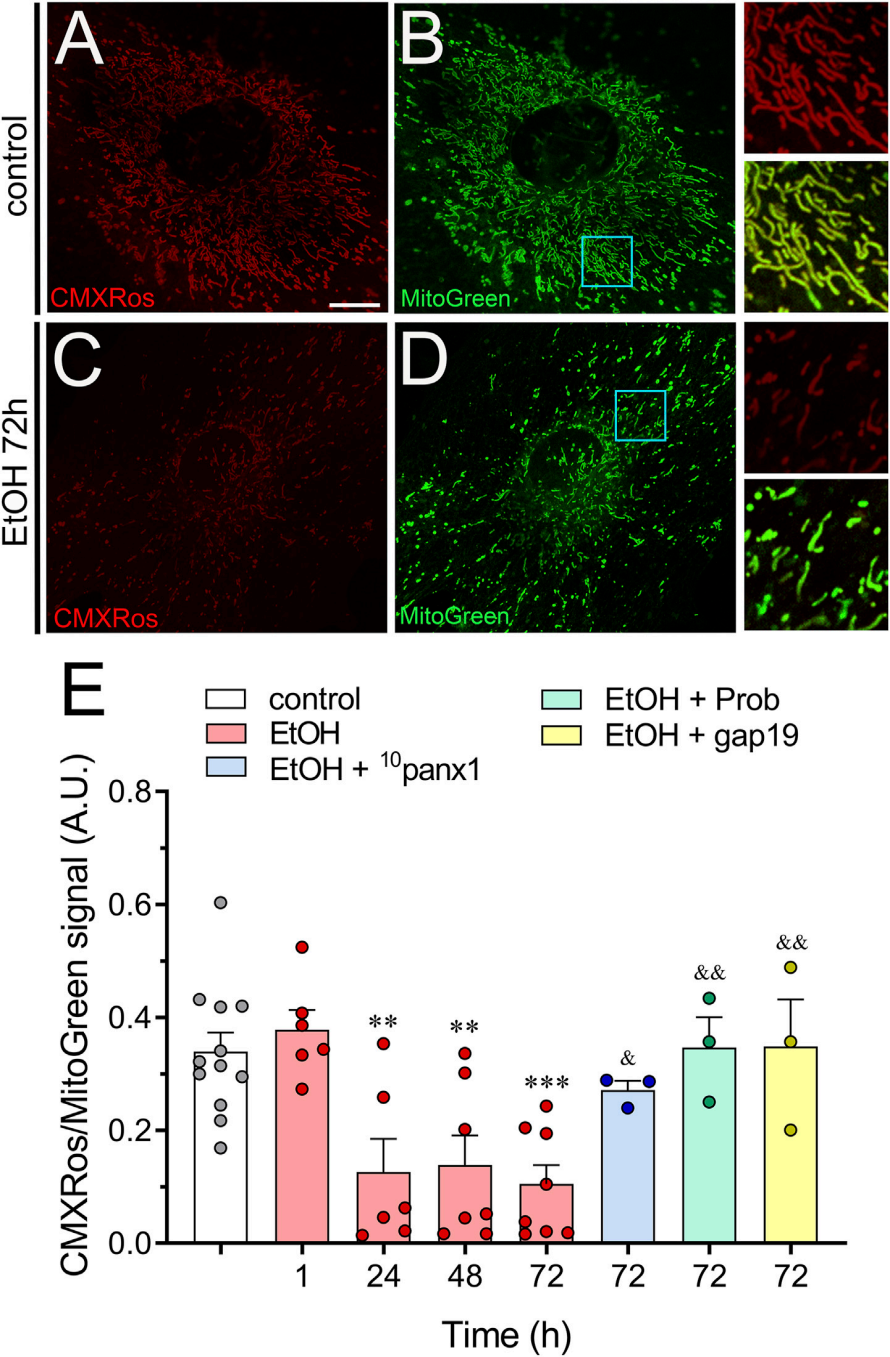
Ethanol decreases mitochondrial membrane potential via the activation of Panx1 and Cx43 hemichannels in astrocytes. **(A–D)** Representative photomicrographs of MitoRed CMXRos (red) and MitoGreen (green) fluorescence by astrocytes under control conditions **(A, B)** or following the treatment with 100 mM ethanol for 72 h **(C,D)**. Insets: × 2.5 magnification of the indicated area of panels **(B, D)**. **(E)** Averaged data of MitoRed CMXRos normalized to MitoGreen fluorescence by astrocytes under control conditions (white violin) or treated for several time periods with 100 mM ethanol alone (red violin). Also, it is shown the effect of the following pharmacological agents in astrocytes treated with 100 mM ethanol for 72 h: 100 µM ^10^panx1 (blue violin), 500 µM probenecid (Prob, green violin) or 100 µM gap19 (yellow violin). **p < 0.01, ***p < 0.001; ethanol compared to control conditions; ^&^ *p* < 0.05, ^&&^ *p* < 0.01; effect of pharmacological agents compared to ethanol treatment (one-way ANOVA followed by Tukey’s *post hoc* test). Each dot represents an independent culture experiment from a different litter of animals. The value reflects the mean of at least three coverslip replicates, with a minimum of 60 cells analyzed per coverslip. Calibration bar: 10 μm.

The electrochemical H^+^ motive force (Δp) plays a vital role in ATP production and regulation (Zorova et al., 2018). Within Δp, the ΔΨ_m_ component is crucial for sequestering Ca^2+^ and controlling the production of ROS. This makes ΔΨ_m_ a key factor in maintaining cell health (Nicholls, 2004). During periods of cellular stress, changes in [Ca^2+^]_i_ or K^+^ levels can disrupt ΔΨ_m_, potentially compromising Δp and leading to a decline in ATP production (Nicholls and Ward, 2000). In this context, we explored whether ethanol could affect ΔΨ_m_ via the activation of hemichannels by measuring the fluoresce intensity of MitoTracker™ Red CMXRos (CMXRos). CMXRos is a lipophilic cationic fluorescent dye that is concentrated inside mitochondria by their negative ΔΨ_m_ (Pendergrass et al., 2004). Given that CMXRos fluorescence in the cell is increased by both increased ΔΨ_m_ and increased mitochondrial mass density per unit area (Pendergrass et al., 2004), we used MitoGreen staining for controlling changes in mitochondrial mass. Control astrocytes exhibited a strong CMXRos/MitoGreen fluorescence (Figures 6A, B). However, ethanol caused a time-dependent significant decline in the CMXRos/MitoGreen fluorescence, peaking at approximately 72 h of treatment (Figures 6C–E). Importantly, inhibition of Panx1 hemichannels or Cx43 hemichannels strongly counteracted the reduction in CMXRos/MitoGreen fluorescence triggered by the treatment with ethanol for 72 h (Figure 6E). Overall, these findings underscore the involvement of Panx1 and Cx43 hemichannels in the decreased ΔΨ_m_ elicited by ethanol in astrocytes.

## 4 Discussion

In this study, we present the first evidence indicating that ethanol alters Ca^2+^_m_ dynamics and triggers fragmentation of mitochondrial networks in astrocytes. Importantly, these ethanol-induced mitochondrial abnormalities result from the activation of Panx1 and Cx43 hemichannels and are associated with heightened production of mitochondrial superoxide and a decrease in ΔΨ_m_ in astrocytes. In previous work, we demonstrated that ethanol activates Cx43 and Panx1 hemichannels in astrocytes via Toll-like receptor 4, leading to the release of IL-1β and TNF-α and subsequent activation of p38 MAPK and iNOS (Gomez et al., 2024). This process increases the release of gliotransmitters, potentially negatively affecting astroglial survival. However, the impact of ethanol-induced hemichannel activation on mitochondrial function has not been explored until the current study.

Maintaining proper Ca^2+^ levels in mitochondria is crucial for cell survival. Sufficient Ca^2+^ uptake by mitochondria is necessary to activate Ca^2+^-sensitive citric acid cycle enzymes and sustain ATP production (Denton, 2009; Rossi et al., 2019; D’Angelo et al., 2023). Additionally, reductions in Ca^2+^_m_ levels have been proposed as a signal that precedes apoptotic pathways (Zhu et al., 1999). Consistent with this, time-lapse recordings of Rhod-2 fluorescence showed that ethanol reduces basal Ca^2+^_m_ levels compared to control conditions, particularly following 1 or 24 h of treatment. Notably, blocking Cx43 or Panx1 hemichannels suppressed the decrease in basal Ca^2+^_m_ evoked by 1 h, but not 24 h, of ethanol treatment. In fact, inhibition of Panx1 hemichannels strengthened the 24-hour ethanol-induced reduction in basal Ca^2+^_m_. These results are intriguing and could be explained by the specific downstream effects of the autocrine molecules released via hemichannels. Cx43 and Panx1 hemichannels are permeable to Ca^2+^, and an increase in [Ca^2+^]_i_ leads to their activation (De Bock et al., 2012; Fiori et al., 2012; Lopez et al., 2021; Salgado et al., 2024). We recently demonstrated that 24 h of ethanol treatment specifically induces the release of glutamate via Cx43 hemichannels and the release of ATP through Panx1 hemichannels, but not *vice versa* (Gomez et al., 2024). Further studies are needed to determine whether the opposing effects on basal Ca^2+^_m_ homeostasis by Panx1 hemichannels are due to species-specific downstream effects.

Overload of Ca^2+^_m_, often caused by excessive Ca^2+^ transfer from the ER, can lead to the loss of ΔΨ_m_, the release of cytochrome C, and ultimately apoptosis (Hajnoczky et al., 2006). Here, we found that ethanol boosts Ca^2+^_m_ upon ER stress with thapsigargin in astrocytes. By inhibiting SERCA pumps, thapsigargin leads to Ca^2+^ depletion from the ER, forcing mitochondria to take up Ca^2+^ (Lytton et al., 1991; Treiman et al., 1998). Ethanol-treated astrocytes exhibited an exacerbation rather than a lack of Ca^2+^_m_ response to thapsigargin, suggesting a phenomenon of sensitization. Interestingly, this sensitization response did not occur following the blockade of Cx43 and Panx1 hemichannels. Mounting evidence indicates that thapsigargin-induced depletion of ER Ca^2+^ stores, triggers store-operated Ca^2+^ entry (SOCE) via the ER Ca^2+^ sensor STIM1 and the Ca^2+^ channel Orai1 (Antigny et al., 2011). Cx43 and Panx1 hemichannels are permeable to Ca^2+^, and an increase in [Ca^2+^]_i_ leads to their activation (De Bock et al., 2012; Fiori et al., 2012; Lopez et al., 2021; Salgado et al., 2024). Therefore, it is possible that hemichannel-dependent sensitization of thapsigargin-induced Ca^2+^_m_ dynamics is due to thapsigargin increasing the activity of these channels. Supporting this idea, we observed that thapsigargin induced a significant increase in Etd uptake compared to basal conditions in astrocytes treated with ethanol but not in control astrocytes. Notably, when experiments were conducted in the absence of extracellular Ca^2+^, the thapsigargin-induced increase in Etd uptake did not occur, indicating that Ca^2+^ entry from the extracellular space, likely via SOCE, is crucial for this phenomenon.

In pathological conditions characterized by cellular [Ca^2+^]_i_ overload, especially when coupled with oxidative stress, the uptake of Ca^2+^_m_ may initiate pathological processes that culminate in cell death (Duchen, 2000). Interestingly, the ethanol-induced alterations on basal and thapsigargin-mediated Ca^2+^_m_ dynamics were accompanied by enhanced production of mitochondrial superoxide, as assessed by MitoSox staining. These data suggest that ethanol alters Ca^2+^_m_ homeostasis under stressing conditions and mitochondrial-dependent balance of ROS production with potentially damaging consequences for astroglial function and survival. Consistent with this idea, the timeframe during which we observed increased Ca^2+^_m_ levels and superoxide production aligns with the period in which ethanol induces astroglial cell death (Gomez et al., 2024). Further studies with additional Ca^2+^ modulators beyond thapsigargin are needed to uncover the mechanism behind impaired Ca^2+^_m_ homeostasis in ethanol-treated astrocytes. However, the involvement of hemichannels appears to be critical. The mimetic peptide gap19, renowned for its capability to counteract Cx43 hemichannel opening (Iyyathurai et al., 2013), notably reduced ethanol-induced increase in mitochondrial superoxide and thapsigargin-mediated Ca^2+^_m_ dynamics. Additionally, similar inhibitory effects were observed with Panx1 hemichannel blockade using ^10^panx1 or probenecid, highlighting the significant contributions of both Cx43 and Panx1 hemichannels in these responses. However, the pharmacological interpretation of our study should be approached with caution. A recent study by Lissoni et al. (2023) has highlighted potential cross-inhibitory effects between gap19 and ^10^panx1 on hemichannels. Their research demonstrated that ^10^panx1 at concentrations between 100 and 500 µM inhibits Cx43 hemichannels, while 500 µM of gap19 is needed to observe some inhibition of Panx1 hemichannels. Despite this, previous research has shown that ion and molecule permeation in hemichannels can be uncoupled and differentially regulated (Hansen et al., 2014a; Hansen et al., 2014b; Gaete and Contreras, 2020). Further studies are needed to determine whether these peptides’ cross-effects on Cx43 and Panx1 hemichannels also impact molecule permeation.

Mitochondrial fission and fusion govern mitochondrial size, shape, number, and distribution under normal and disease conditions (Chan, 2020; Adebayo et al., 2021). Furthermore, the dynamics of mitochondrial fission and fusion allow mitochondria to meet energy demands and maintain a proper redox balance (Adebayo et al., 2021). We and others have shown that ethanol disrupts ΔΨ_m_ and ATP production while also affecting key proteins involved in mitochondrial fusion and fission in neurons (Tapia-Rojas et al., 2018; Lim et al., 2020; Quintanilla et al., 2020; Liu et al., 2022). These include dynamin-related protein 1, fission protein 1, optic atrophy 1, and mitochondrial fusion protein mitofusin 1. Consistent with this evidence, we found that ethanol reduced mitochondrial network complexity and increased astrocyte mitochondrial fragmentation. Furthermore, our data indicate that ethanol induces a time-dependent decrease in ΔΨ_m_, as measured by CMXRos staining. Notably, blocking Panx1 or Cx43 hemichannels totally counteracted these ethanol-induced mitochondrial abnormalities in astrocytes. Ca^2+^_m_ overload leads to mitochondrial fragmentation (Tsujimoto et al., 2006) and the opening of the non-selective, high-conductance mitochondrial permeability transition pore (mPTP) in the inner mitochondrial membrane (IMM). The mPTP allows solutes with molecular mass up to 1.5 kDa to pass through, compromising energy metabolism and mitochondrial structural integrity through osmotic swelling (Tsujimoto et al., 2006). Notably, a recent study found that blocking Cx43 hemichannels reduces mitochondrial swelling caused by pro-inflammatory cytokines (Okolo et al., 2024). The interaction between hemichannels and mitochondria appears to be reciprocal. Indeed, the decrease in ΔΨm evoked by metabolic inhibition precedes the opening of hemichannels (Sanchez et al., 2009). This crosstalk may involve hemichannels located specifically at the mitochondria rather than on the plasma membrane. Both Cx43 and Panx1 have been detected in mitochondria (Miro-Casas et al., 2009; Rusiecka et al., 2023), and recent research suggests that Cx43 hemichannels in the mitochondria regulate ATP generation by mediating K^+^, H^+^, and ATP transfer across the IMM. This interaction, facilitated by the close association of Cx43 hemichannels with mitochondrial ATP synthase, helps maintain mitochondrial redox levels in response to oxidative stress (Zhang et al., 2022). Further research is needed to clarify the impact of mitochondrial hemichannels *versus* those in the plasma membrane on ethanol-induced impairment on mitochondrial function and morphology.

It is important to note that 100 mM ethanol, the concentration used here, aligns with concentrations used in seminal studies by leading experts examining ethanol’s impact on cultured astrocytes (Ritchie et al., 1988; Kimelberg et al., 1993; Yagle and Costa, 1999; Aschner et al., 2001; Adermark et al., 2004; Blanco et al., 2004). Individuals with alcohol use disorder can exhibit blood alcohol concentrations (BACs) exceeding 0.55% (∼120 mM) while still performing tasks (Adachi et al., 1991). During binge drinking, they may maintain elevated BAC levels for extended periods despite ongoing alcohol metabolism. Therefore, while the concentrations used in this study may not be directly relevant to alcohol intoxication in healthy individuals, they provide insights into the cellular mechanisms underlying alcohol-induced brain damage in binge-drinking alcoholic individuals (Didier et al., 2023). Interestingly, acetaldehyde, a direct metabolite of ethanol oxidation, contributes to many behavioral effects of ethanol (Quertemont et al., 2004; Vasiliou et al., 2006). Due to the abundance of aldehyde dehydrogenases in the blood-brain barrier, acetaldehyde formed outside the central nervous system cannot enter it (Deitrich et al., 1989; Zimatkin, 1991). Thereby, its concentration in the brain is primarily determined by local production from ethanol. Given the existence of ethanol-oxidizing pathways in the brain (Heit et al., 2013), where astrocytes may participate (Sarc et al., 2011), we cannot rule out the possible involvement of ethanol metabolites in our system. Future studies are necessary to elucidate the effect of acetaldehyde and other ethanol derivatives, such as acetate, on astroglial hemichannel activity.

The mechanisms behind the hemichannel-mediated mitochondrial alterations in ethanol-treated astrocytes could be multiple. We hypothesize that ethanol-mediated opening of hemichannels in astrocytes leads to an unregulated influx of potentially harmful substances like Ca^2+^. Since hemichannels allow the passage of Ca^2+^ and their activity is regulated by [Ca^2+^]_i_ (Schalper et al., 2010; De Bock et al., 2012; Fiori et al., 2012; Lopez et al., 2021; Salgado et al., 2024), it is plausible that their opening might trigger an overload of [Ca^2+^]_i_, subsequently disrupting crucial functions vital for astrocyte survival, including Ca^2+^_m_ and antioxidant defenses (Stephen et al., 2014). Moreover, the uncontrolled influx of Na^+^ and chloride ions Cl^−^ through hemichannels could induce osmotic and ionic imbalances (Paul et al., 1991; Diaz et al., 2019), a phenomenon that impairs mitochondrial function and ATP production (Nunes et al., 2015). The data obtained here supports the notion that ethanol directly affects the function of mitochondria in astrocytes by a mechanism that involves the activation of Panx1 and Cx43 hemichannels. Our findings indicate tha ethanol transitorily alters Ca^2+^_m_ dynamics and morphological features of mitochondria within a timeframe of 1–48 h post-treatment. This harmonizes with the fact that ethanol-induced activation of astroglial hemichannels lasts no more than 24 h (Gomez et al., 2024). Although these responses are temporary, they are sufficient to cause a persistent reduction in mitochondrial membrane potential for at least 72 h. We speculate that exacerbated hemichannel activity evoked by ethanol may impair [Ca^2+^]_i_ homeostasis, stressing Ca^2+^_m_ with potentially negative consequences for mitochondrial fusion and fission dynamics and astroglial bioenergetics. Grasping this mechanism presents a hopeful path toward creating new pharmacological strategies aimed at protecting astrocyte integrity and enhancing neuronal resilience against the multifaceted challenges posed by different alcohol use disorders.

## Data Availability

The original contributions presented in the study are included in the article/Supplementary Material, further inquiries can be directed to the corresponding author.
